# Increased Interfacial Area between Dielectric Layer and Electrode of Triboelectric Nanogenerator toward Robustness and Boosted Energy Output

**DOI:** 10.3390/nano9010071

**Published:** 2019-01-06

**Authors:** Donghyeon Yoo, Eun Yeong Go, Dongwhi Choi, Jeong-Won Lee, Insang Song, Jae-Yoon Sim, Woonbong Hwang, Dong Sung Kim

**Affiliations:** 1 Department of Mechanical Engineering, Pohang University of Science and Technology (POSTECH), 77 Cheongam-ro, Pohang, Gyeongbuk 790-784, Korea; ta2two@postech.ac.kr (D.Y.); go7064@postech.ac.kr (E.Y.G.); aaron@postech.ac.kr (J.-W.L.); whwang@postech.ac.kr (W.H.); 2 Department of Mechanical Engineering, Kyung Hee University, 1732, Deogyeong-daero, Giheung-gu, Yongin-si, Gyeonggi-do 17104, Korea; dongwhi.choi@khu.ac.kr; 3 Agency for Defense Development (ADD), Yuseong, Daejeon 305-600, Korea; energysong@add.re.kr; 4 Department of Electronic and Electrical Engineering, Pohang University of Science and Technology (POSTECH), 77 Cheongam-ro, Pohang, Gyeongbuk 790-784, Korea; jysim@postech.ac.kr

**Keywords:** robust triboelectric nanogenerator, increased interfacial area, microstructured Al electrode by wet etching in HCl, long-lasting performance

## Abstract

Given the operation conditions wherein mechanical wear is inevitable, modifying bulk properties of the dielectric layer of a triboelectric nanogenerator (TENG) has been highlighted to boost its energy output. However, several concerns still remain in regards to the modification due to high-cost materials and cumbersome processes being required. Herein, we report TENG with a microstructured Al electrode (TENG_ME) as a new approach to modifying bulk properties of the dielectric layer. The microstructured Al electrode is utilized as a component of TENG to increase the interfacial area between the dielectric layer and electrode. Compared to the TENG with a flat Al electrode (TENG_F), the capacitance of TENG_ME is about 1.15 times higher than that of TENG_F, and the corresponding energy outputs of a TENG_ME are 117 μA and 71 V, each of which is over 1.2 times higher than that of the TENG_F. The robustness of TENG_ME is also confirmed in the measurement of energy outputs changing after sandpaper abrasion tests, repetitive contact, and separation (more than 10^5^ cycles). The results imply that the robustness and long-lasting performance of the TENG_ME could be enough to apply in reliable auxiliary power sources for electronic devices.

## 1. Introduction

With the advent of the Internet of Things (IoT)—beyond miniaturized and wearable electronic devices—the ubiquitous and persistent supply of energy needing to be anywhere has been a critical issue. The issue becomes even more prominent in isolated circumstances such as mountain and industrial regions due to the limited lifetime of batteries [1,2]. Furthermore, the large capacity battery has a compromise for a long lifetime in its heavy weight. To overcome such limitations, energy harvesting technologies have been proposed as auxiliary sources or substitutions of the conventional energy supply system. Among them, the triboelectric nanogenerator (TENG), which is operated by the combination of contact electrification and electrical induction, is attracting much attention due to its high accessibility and fast-growing efficiency [3,4,5,6,7]. The basic structure of the TENG is composed of a dielectric layer where the frictional charges are generated after contact electrification and an electrode where the induced charges are located by electrical induction. To enhance the energy output of the TENG, several strategies have been focused on modulating the properties of the dielectric layer. As a part of them, the previous works reported the modification of surface properties of the dielectric layer to increase surface charge density, such as introducing surface topography, chemical coating, and injecting ions into the dielectric layer [8,9,10,11]. However, the degradation of energy output is inevitable because of the mechanical wear of the surface topography and chemical coating, coming from the contact and separation of the dielectric layer with a counter layer. To alleviate it, a new approach of modifying bulk properties of the dielectric layer while excluding imposing surface topography has been suggested, which includes aligning dipole moment and increasing compressibility or capacitance of the dielectric layer [12,13,14,15,16]. Among them, increasing the capacitance of the dielectric layer has attracted much attention recently because the energy output of the TENG is not only boosted, but long-lasting [17,18,19,20,21]. Generally, the capacitance of the dielectric layer can be increased by filling the layer with materials of a high dielectric constant. Thus, as the friction continues, more frictional charges can be accumulated in the dielectric layer, which can eventually boost the energy output of the TENG by allowing more induced charges to be located in the electrode [19,22]. However, previous approaches have limitations in view of fabrication because expensive materials are required to meet the conditions of filling materials, and cumbersome processes are inevitably carried out considering the misalignment or overdose of the materials can degrade energy output [14,16,20,21]. For the above reasons, developing a cost-effective and simple method to increase the capacitance of the dielectric layer could greatly contribute to promoting the practicality of the long-lasting TENG.

In this study, we first propose the triboelectric nanogenerator with a microstructured aluminum electrode (TENG_ME), which increases the interfacial area between the dielectric layer and the electrode, thereby in turn achieving a facile increase of the capacitance of the dielectric layer. The microstructures of the electrode can be easily fabricated by physical or chemical methods such as metal casting, machining, rubbing with sandpaper, and dry/wet etching. Among them, wet etching in HCl is selected in this study due to its versatility for various metals such as aluminum, copper, and titanium [23,24,25]. Specifically, the wet-etched aluminum is chosen as an electrode of the TENG considering its cost-effectiveness and high controllability [26,27,28]. The advantageous effect of using the microstructured Al electrode for the purpose of electrical performance of the TENG_ME is successfully confirmed by investigating both the enhanced capacitance of the dielectric layer and the boosted energy output of the TENG_ME. The TENG_ME shows superiority to mechanical wear in harsh environments and long-lasting performance compared to the conventional TENGs with surface topography on its dielectric layer. Considering the mechanical wear problem is raised as a big issue to achieve robust operation of the TENG, the present study would bring it one step closer to a faithful auxiliary power source for electronic devices.

## 2. Materials and Methods

### 2.1. Fabrication of the TENGs

The Al electrode (5052, Hyundae Metal, Chungcheongbuk-do, Korea) and 4.5 μm thick polyvinylidene fluoride film (PVDF, Hanarotr, Gyeonggi-do, Korea) are utilized as an electrode and a dielectric layer of the TENGs, respectively. Polyimide (PI, 4science, Gyeonggi-do, Korea) is utilized as a counter layer. The flat Al electrode is fabricated by the electropolishing of a 3 cm by 3 cm Al substrate in 7 °C perchloric acid/ethanol (60/40 vol. %) for 3 min, applying a constant voltage of 20 V, which was a well-established condition in the previous work [29]. The microstructured Al electrode is fabricated by wet etching of the same Al substrate in 2 M HCl solution for 5 min at room temperature after cleaning in a NaOH solution for 1 min at room temperature. To achieve stable adhesion between PVDF and the Al electrode, the PVDF films are coated in 3-aminopropyl triethoxysilane (99% APTES, Sigma-Aldrich, St. Louis, MO, USA)/ethanol/DI water (5/90/5 vol. %) after oxygen plasma treatment [30]. Five sheets of PVDF film are attached to the as-prepared Al electrodes using a hot embossing process, which is conducted under a pressure of 5 MPa and a temperature of 180 °C. The stable integration of PVDF films and the Al electrode are confirmed by the image from a focused ion beam (Helios 650, FEI, Hillsboro, OR, USA) (Figure S1 in Supplementary Materials).

The surface topography on a PVDF film is fabricated by the hot embossing process with a stamp possessing reverse micropyramid surface structures under the same hot embossing process conditions. The fabrication procedure of the stamp begins with anisotropic chemical etching of a silicon wafer (P-type (100) test grade, 4science, Gyeonggi-do, Korea) into micropyramid structures. The mixture of KOH/DI water/IPA (20/77/3 wt %) is used as the etching solution, and the etching temperature and time are 70 °C and 15 min, respectively [31]. Thereafter, the surface structures of the etched wafer are replicated by a polydimethylsiloxane (PDMS, Dow Corning, Midland, MI, USA) replica molding process. As a result, the PDMS replica has the reverse micropyramid surface structures, which is utilized as the stamp in the hot embossing process.

### 2.2. Evaluation of TENG Characteristics

The capacitance of the TENG is measured by LCR meter (E4980AL, Keysight, Santa Rosa, CA, USA) by changing an AC signal frequency from 20 Hz to 10 kHz at a fixed voltage of 1 V. In order to evaluate the available energy, the energy output of the TENG is measured by connecting a conventional rectifier with the TENG. The rectified open-circuit voltage (*V_OC_*) is measured by using an oscilloscope (DS1074z, Rigol, Beijing, China), and the rectified short-circuit current (*I_SC_*) is measured by using the oscilloscope connected to a pre-amplifier (SR570, Standard Research Systems). The energy output is expressed by the representative values, which are chosen by averaging each peak value of *I**_SC_*** and *V**_OC_*** within a certain time interval. All of the experiments related to the energy output measurements of the TENG are conducted at an ambient temperature of 26 ± 0.5 °C and a relative humidity of 60 ± 5%. The surface morphology of both flat Al electrode and microstructured Al electrode are examined using field emission scanning electron microscopy (FE-SEM) (JSM 7401F, JEOL, Akishima, Japan) and 3D profiler (Wyko NT1100, Veeco Touson, Tucson, AZ, USA). For the evaluation of robustness, a sandpaper abrasion test is conducted. In the test, TENGs are attached to a 100 g weight (1.1 kPa) and then moved back and forth 300 cm while facing the sandpaper (particle size #400, Smato, Gyeongsangnam-do, Korea).

## 3. Results and Discussion

### 3.1. Expected Performance of the TENGs

**Figure 1:**
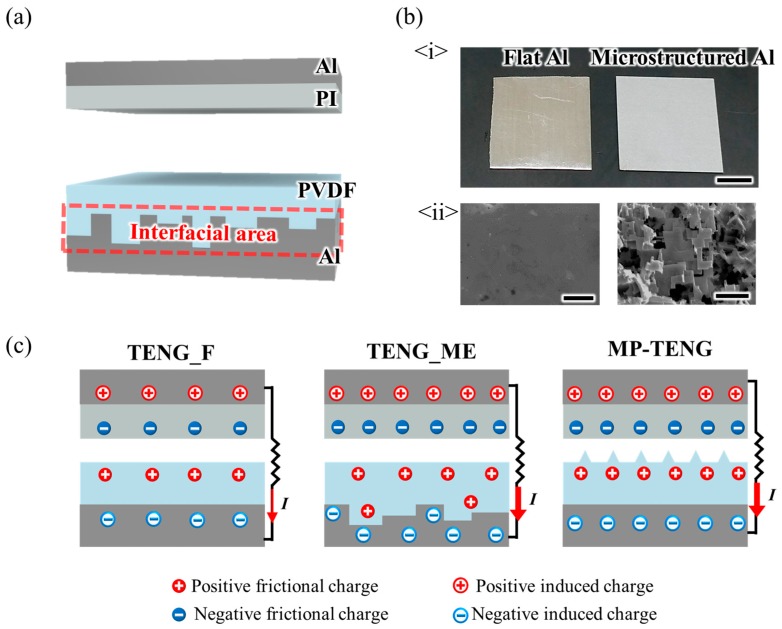
Fabrication of the TENG with a flat aluminum (Al) electrode (TENG_F), the TENG with a microstructured Al electrode (TENG_ME), and the TENG consisting of a flat Al electrode and a dielectric layer with micropyramid surface topography (MP-TENG). (**a**) Schematic diagram of polyimide (PI) counter layer and the TENG_ME which is composed of the polyvinylidene fluoride (PVDF) film and the microstructured Al electrode. (**b**) <i> Images of a flat Al electrode and a microstructured Al electrode. The scale bar is 1 cm. <ii> The scanning electron microscopy (SEM) images of the flat Al electrode and the microstructured Al electrode. The scale bar is 5 μm. (**c**) Expected difference between TENG_F, TENG_ME, and MP-TENG.

In this work, all of the TENGs consist of a dielectric layer which is attached to an Al electrode. PVDF is utilized as a material for a dielectric layer due to its outstanding ability to attract electrons. As a counter layer, polyimide (PI, 4science, Gyeonggi-do, Korea) is utilized due to its high tendency to be positively charged after sequential contact and separation with PVDF as shown in Figure 1a [32]. The operation mechanism of the TENGs in this work is as follows. When contact and separation between the PI counter layer and the PVDF dielectric layer occur in a vertical contact mode, the frictional charges are generated so that PI becomes positively charged and PVDF is negatively charged. The electrostatic equilibrium between PI and PVDF is broken as the relative distance between them increases. Thereafter, the counter charges are induced between both electrodes which are attached to each of the PI and PVDF layers to satisfy a new electrostatic equilibrium. As a result, an electric current is generated from the movement of the induced charges so the energy outputs of the TENGs can be measured. Since both PI and PVDF are dielectric materials, the present TENGs could be considered as a kind of capacitor. Thus, if contact and separation continue, the frictional charges in each PI and PVDF start to be accumulated and the amount of the frictional charges reaches saturation. It implies that the number of induced charges and the corresponding energy output also increase to certain saturation points. Considering that the capacitance (*C*) of the dielectric material indicates the ability to accumulate the charges, the increased *C* of PVDF results in enhancing the maximum amount of induced charges in the connected electrode followed by the boosted energy output of a TENG. Meanwhile, it is well known that the *C* of the dielectric materials increases as the interfacial area between dielectric materials and electrode increases because the surplus area acts as an additional capacitor [33,34]. On the basis of the advantageous effect of increased interfacial area, a new strategy to boost energy output of a TENG was recently reported as utilizing coarse carbon tape as the electrode [35]. However, in previous work, Al was utilized as the electrode of the control TENG group, which means the variable control of the electrode material is improperly achieved. Considering the fact that the material property of the electrode can affect the decay of the frictional charges, the previous work could not accurately identify the effect of increased interfacial area on the energy output of TENG [36]. In this work, the interfacial area of the TENG is increased from the fabrication of the microstructured Al electrode by wet etching of the Al substrate in HCl solution. Due to the unity of electrode material, the accurate study on the effect of increased interfacial area on the energy output can be assured. Figure 1b-<i> shows that the flat Al electrode has a mirror-like surface, whereas the microstructured Al electrode is rather opaque due to the presence of the surface microstructures. According to the surface roughness measurements from the 3D profiler, the root-mean-square roughness (*R_q_*) of the microstructured Al electrode is 5.91 μm, which is about 40 times higher than that of a flat Al electrode (Figure S2 in Supplementary Materials). Figure 1b-<ii> shows the corresponding scanning electron microscopy (SEM) images.

Introducing surface topography onto a dielectric layer is a common strategy to boost the energy output of the TENG [37,38,39,40]. Given the fact that the TENG is operated under numerous physical contact and separation conditions, the reliable electrical performance for long-lasting operation is hard to be expected from the surface topography-based strategy because mechanical wear of the surface structure is inevitable. In contrast, a robust TENG with reliable electrical performance could be expected in the present strategy because the microstructures of the electrode are located inside the TENG and free from mechanical wear.

To confirm the abovementioned expected performance, TENG with a flat Al (TENG_F), TENG with a microstructured Al (TENG_ME), and TENG consisting of a flat Al and a dielectric layer with micropyramid surface topography (MP-TENG) are fabricated. Figure 1c shows a schematic diagram of all of the TENGs.

### 3.2. Electrical Characteristics of the TENG_F and TENG_ME

**Figure 2:**
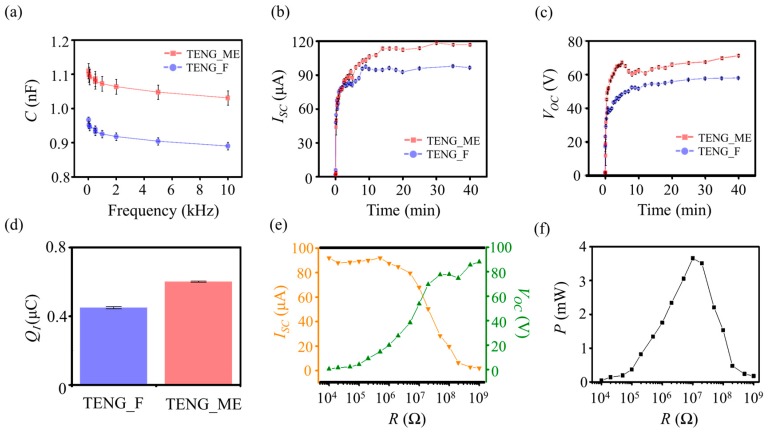
Energy outputs of TENGs. (**a**) Capacitance (*C*) of the TENG with a microstructured Al electrode (TENG_ME) and the TENG with a flat Al electrode (TENG_F). (**b**) The rectified short-circuit current (*I_SC_*) of the TENG_ME and the TENG_F. (**c**) The rectified open-circuit voltage (*V_OC_*) of the TENG_ME and the TENG_F. (**d**) The amount of the induced charges (*Q_I_*) of the TENG_ME and the TENG_F. (**e**) The effect of load resistance (*R*) on *I_SC_* and *V_OC_* of the TENG_ME. (**f**) The effect of *R* on instantaneously generated electric power (*P*) of the TENG_ME.3.3. Robustness of the TENG_ME.

The effect of the increased interfacial area on the TENG is first confirmed by comparison of capacitance (*C*) of the TENG_F and the TENG_ME. As expected, Figure 2a shows that the *C* of the TENG_ME is about 1.15 times higher than *C* of the TENG_F for the AC signal frequencies from 20 Hz to 10 kHz. The corresponding energy outputs are examined by *I_SC_* and *V_OC_* under a fixed vertical contact and separation condition (a frequency of 10 Hz and an applied force of 15 N). Figure 2b,c show the saturation behaviors of *I_SC_* and *V_OC_* over time, respectively. In the early stage, both *I_SC_* and *V_OC_* have a tendency to increase since the frictional charges are accumulated in both PI and PVDF as explained in previous reports [19,22]. After 40 min of its operation, *I_SC_* and *V_OC_* of the TENG_ME saturate to 117 μA and 71 V, respectively, each of which is over 1.2 times higher than those of the TENG_F. The similarity in the increasing rate of *C* and the energy output implies a positive correlation between them. Furthermore, a positive relationship between the energy output and the interfacial area of the TENG could be observed. Thus, more comprehensive studies on the effect of the interfacial area between the dielectric layer and electrode on the electrical performance of TENG are required, such as tuning the surface topography (structure size and geometry) of the electrode, to completely establish the strategy for boosting energy output of a TENG.

By integrating *I_SC_* over the operation time, the amount of the induced charges (*Q_I_*) is calculated to confirm our hypothesis of boosted charge accumulation as depicted in Figure 1c. Figure 2d shows *Q_I_* of the TENG_F and the TENG_ME at their saturation states. *Q_I_* of the TENG_ME is about 0.6 μC, which is 1.5 times higher than the TENG_F. From the above experimental results and analysis, the present work incorporating the increase of interfacial area between the dielectric layer and the electrode can be a new strategy for boosting the energy output of the TENG.

For the further characterization of TENG energy output, the effect of triggering frequency on the *V_OC_* is investigated. Under the frequencies from 1 Hz to 10 Hz, the *V_OC_* increases from 54 V to 71 V, which is the similar tendency with previous results (Figure S3 in Supplementary Materials) [41,42]. Furthermore, the internal electrical impedance of the TENG_ME, the effect of load resistance (*R*) on instantaneously generated electric power (*P*), defined by multiplying *I_SC_* and *V_OC_*, is also investigated. Figure 2e shows that *V_OC_* increases up to 88 V as *R* increases, whereas *I_SC_* trend is reversed as previously reported, decreasing down to 2 μA [8,9,10,11,12,13,14,15,16,17,18,19,20,21]. As a result, a maximum *P* is found to be 3.5 mW when *R* is 10 MΩ, which implies that an internal electrical impedance of the present TENG_ME is about 10 MΩ (Figure 2f).

**Figure 3:**
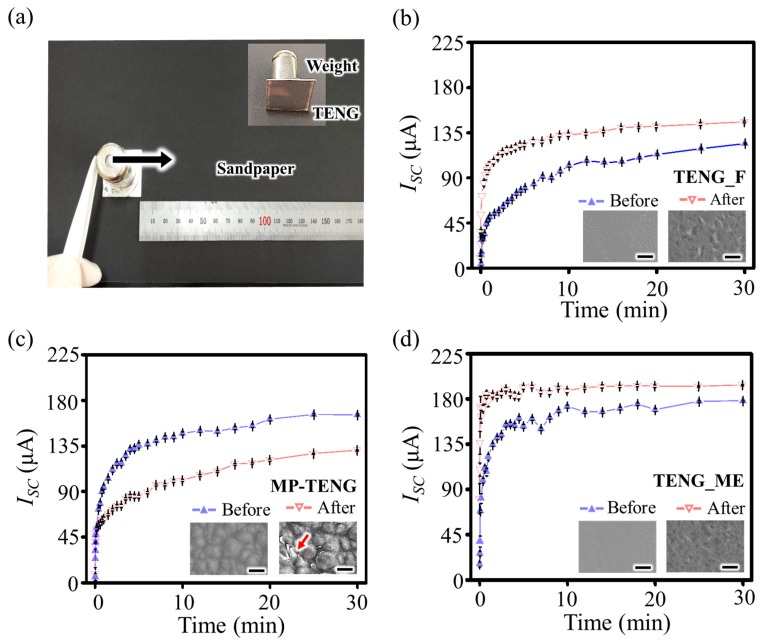
Robustness evaluation of the TENGs. (**a**) Image of sandpaper abrasion test. (**b**) The rectified short-circuit current (*I_SC_*) change of the TENG with a flat Al electrode (TENG_F). The inset shows the scanning electron microscopy (SEM) image of TENG_F before and after sandpaper abrasion test. The scale bar is 5 μm. (**c**) *I_SC_* change of the TENG consisting of a flat Al electrode and a dielectric layer with micropyramid surface topography (MP-TENG). The inset shows scanning electron microscopy (SEM) image of MP-TENG before and after sandpaper abrasion test. The scale bar is 10 μm. (**d**) *I_SC_* change of the TENG with a microstructured Al electrode (TENG_ME). The inset shows the scanning electron microscopy (SEM) image of TENG_ME. The scale bar is 5 μm.

In this study, the sandpaper abrasion test is carried out to artificially generate mechanical wear, and then the change in surface structures and the corresponding energy output of the TENG is investigated to verify the robustness of the present TENG_ME (Figure 3a). In this test, three different types of TENGs, i.e., TENG_ME, TENG_F, and MP-TENG are used. Under the cyclic force with a magnitude of 50 N in a frequency of 10 Hz, the change in *I_SC_* of each TENG is observed during 30 min of contact and separation. Figure 3b shows that the saturation value of *I_SC_* of the TENG_F increases from 120 μA to 145 μA due to newly formed surface structures on the flat PVDF layer of the TENG_F after rubbing it with sandpaper. The SEM images demonstrate the additional surface structures on the PVDF layer (the inset of Figure 3b). The sandpaper abrasion test is also performed on MP-TENG, which represents the strategy for introducing topography onto a dielectric layer surface. Figure 3c shows the sandpaper abrasion test result on the MP-TENG, indicating that the saturation value of *I_SC_* decreased from 165 μA to 130 μA. The inset of Figure 3c indicates the deterioration of micropyramid surface structures, which means the ability to increase surface charge density after contact and separation is lost due to the reduced contact surface area. In the case of TENG_ME, however, the saturation value of *I_SC_* increases from 178 μA to 193 μA (Figure 3d). That the saturation value of *I_SC_* of the TENG_ME is higher than that of the MP-TENG after the abrasion test implies the present strategy of enlarging the interfacial area could be one effective way to boost the energy output of the TENG. It is noted that the electrical energy output of the TENG_ME increases after rubbing it with the sandpaper, which is similar to the case of the TENG_F due to the additionally formed surface structures on the originally flat PVDF surface by abrasion (the inset of Figure 3d). Long-lasting performance of the TENG_ME is further evaluated under more than 10^5^ cycles of repetitive contact and separation, indicating a stable energy output from the TENG_ME (Figure S4 in Supplementary Materials). Given that the frequency of biomechanical energy, an abundant energy source, ranges from 0.5 Hz to 3 Hz; the long-lasting performance implies that reliable and continuous operation for more than 10 h is expected [43]. The above results confirm the robustness and reliable operation of the TENG_ME, indicating the potential broad applicability of the TENG_ME.

### 3.3. Practical Demonstrations of the TENG_ME

**Figure 4:**
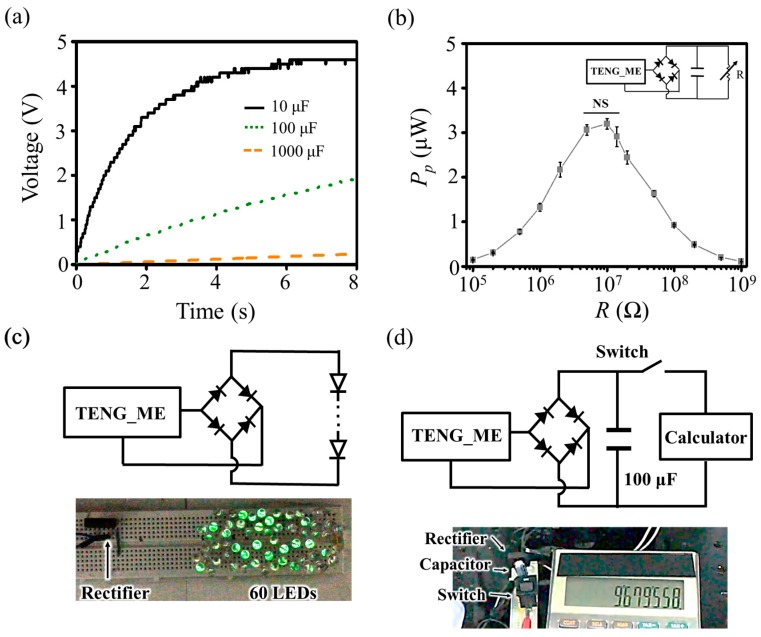
Demonstrations of the the TENG with a microstructured Al electrode (TENG_ME). (**a**) Charging capacitors by the TENG_ME. (**b**) The effect of load resistance (*R*) on practical power (*P_P_*) of the TENG_ME. (**c**) Lightening LEDs by the TENG_ME. (**d**) Operating the conventional electronic calculator by the TENG_ME.

Beyond the energy harvesting ability of the TENG, it is also important to evaluate the actual amount of energy stored or supplied for the practical operation of electronic devices. As part of that, charging conventional capacitors by using the present TENG_ME is demonstrated first. The TENG_ME is connected with a rectifier and the rectified energy is charged to each 10 μF, 100 μF, and 1000 μF capacitor at a fixed contact and separation condition (a frequency of 10 Hz and an applied force of 15 N) as shown in Figure 4a. The result implies that the instantaneous energy output from the TENG_ME can be stored and thus become available to supply stable power. Furthermore, the effect of *R* on practical power (*P_P_*) is studied assuming that the power can be supplied into several electronic devices. In this case, a DC voltage is measured across *R* connected in parallel to the 10 μF capacitor so that practical power is calculated from the relationship of power expressed by the resistance and the voltage across it. As a result of experiments on three TENG_MEs, *P_P_* reaches about 3.2 μW at 10 MΩ as shown in Figure 4b. However, there is no statistical difference (*p* < 0.05) of *P_P_* when *R* is 5 MΩ, 10 MΩ, and 15 MΩ according to the one-way analysis of variance (ANOVA) test. The reason why optimum value of *R* is observed in the range would be attributed to the different electrical characteristics of each TENG_ME. As observed in Figure 2a, the *C* variance of TENG_ME is investigated due to inhomogeneous microstructure of the Al electrode and different composition ratio of the Al electrode (5052 aluminum alloy) such as the ratio of magnesium, iron, chromium, and copper. Meanwhile, it is noted that the impedance value of at the maximum *P_P_* could be lower than the internal electrical impedance (~10 MΩ) discussed in Figure 2f. The reason why the optimum value of *R* is shifted is presumed that the connected 10 μF capacitor acts as another resistor. In general, *R* of the electronic circuit is lower than several kΩ, so that the maximum *P_P_* could not be completely transferred to the electronic devices [44,45,46]. Given that a capacitor can affect the total resistance of the circuit connected to the TENG_ME, the effort to reduce the optimum value of *R* of the TENG_ME would be valuable by intensively investigating the effect of a capacitor on the electrical performance of the TENG. Finally, the energy output of the TENG_ME is also demonstrated by lighting LEDs and powering a conventional electronic calculator. Figure 4c shows the energy output from the TENG_ME is enough to simultaneously light 60 LEDs (Video S1 in Supplementary Materials). Additionally, Figure 4d shows the electronic calculator can be operated after charging the 100 μF capacitor for 15 min, which indirectly shows that the present TENG_ME can supply electricity to power the commercially available portable electronics (Video S2 in Supplementary Materials).

## 4. Conclusions

In this study, a novel strategy to boost the energy output of the TENG is suggested by increasing the interfacial area between the dielectric layer and the electrode, which are main components of the TENG, thereby improving the capacitance of the dielectric layer. To increase the interfacial area, a microstructured Al electrode is fabricated by a simple wet etching process and utilized as an electrode in the TENG_ME. Compared to the TENG_F, the TENG_ME generates boosted energy output thanks to the increased capacitance of the dielectric layer. Additionally, the strategy, which is free from the effect of mechanical wear, successfully alleviates the chronic problem of the degradation of energy outputs frequently observed in the previous works due to the deterioration of the surface structures by mechanical wear. The results of sandpaper abrasion tests and long-lasting operation validate the robustness of the TENG_ME. Comprehensive studies of this work show that the TENG can come closer to being a reliable auxiliary power source.

## Supplementary Materials

The following are available online at http://www.mdpi.com/2079-4991/9/1/71/s1, Figure S1: Focused ion beam image of TENG_ME, Figure S2: 3D profiler images of Al electrodes, Figure S3: The effect of triggering frequency on the *V_OC_* of the TENG_ME, Figure S4: Long-lasting performance of the TENG_ME, Video S1: Lightening LEDs by TENG_ME, Video S2: Operating a conventional electronic calculator by TENG_ME.
